# Lack of Vaccination Against COVID-19, Obesity and Coexistence of Cardiovascular Diseases as Independent Predictors of a Higher Number of ECG Changes in Patients with Previous SARS-CoV-2 Infection

**DOI:** 10.3390/jcm14072329

**Published:** 2025-03-28

**Authors:** Ewelina Beck, Agata Malczyk, Irena Dykiert, Michał Fułek, Katarzyna Fułek, Małgorzata Poręba, Paweł Gać, Rafał Poręba

**Affiliations:** 1 Department of Environmental Health, Occupational Medicine and Epidemiology, Wroclaw Medical University, 50-345 Wroclaw, Poland; 2 Department of Diabetology, Hypertension and Internal Diseases, Institute of Internal Diseases, Wroclaw Medical University, 50-556 Wroclaw, Poland; 3 Division of Pathophysiology, Department of Physiology and Pathophysiology, Wroclaw Medical University, 50-368 Wroclaw, Poland; 4 Department of Otolaryngology, Head and Neck Surgery, Wroclaw Medical University, 50-556 Wroclaw, Poland; 5 Department of Biological Principles of Physical Activity, Wroclaw University of Health and Sport Sciences, 51-612 Wroclaw, Poland

**Keywords:** body mass index, COVID-19, electrocardiography, vaccination

## Abstract

**Objectives.** Many studies have confirmed the existence of a relationship between SARS-CoV-2 virus infection and an increased incidence of arrhythmia in the population of adults, children and adolescents. It is believed that one of the potential side effects of COVID-19 vaccination is arrhythmia. However, large-scale studies confirming the relationship between COVID-19 vaccination and cardiac arrhythmia are currently lacking. The objective of this study was to analyze the occurrence of arrhythmias in 24 h Holter ECG monitoring among patients who had experienced COVID-19, comparing those who were vaccinated against SARS-CoV-2 with those who were unvaccinated. **Methods.** The study was performed on a study group of 237 patients, who underwent 24 h Holter monitoring. **Results.** Ventricular extrasystoles (VEs) were distinctively more common in patients, who had COVID-19 infection and were not vaccinated for COVID-19 comparing to the control group. Similarly, research has shown that supraventricular extrasystoles (SVEs) occurred remarkably more frequently in both unvaccinated and vaccinated patients after COVID-19 infection in relation to control groups. Multivariable regression analysis demonstrates that, in the whole study group, obesity, arterial hypertension, previous myocardial infarction and lack of vaccination against COVID-19 are independent risk factors for higher VE rates. Obesity, diabetes type 2 and lack of vaccination against COVID-19 are independent risk factors for higher SVE rates. The use of β-blockers is an independent protective factor against higher VE and SVE rates, and the use of ACE inhibitors against higher SVE rates. **Conclusions.** In this study, the authors obtained promising results for the future, facilitating further discussion and research on the topic of the antiarrhythmic advantages of COVID-19 vaccination. Moreover, the knowledge acquired in this study serves as a valuable tool for effectively promoting COVID-19 vaccination among patients.

## 1. Introduction

The SARS-CoV-2 virus, responsible for the COVID-19 pandemic, has had far-reaching global implications since its emergence in 2020 [1,2,3]. SARS-CoV-2, classified as an RNA virus within the coronavirus family, primarily causes severe acute respiratory syndrome (SARS) [4]. Beyond its respiratory manifestations, the virus significantly affects cardiovascular function [5].

### 1.1. ACE2 Receptor: Gateway for SARS-CoV-2 and Mediator of Cardiovascular Impact

SARS-CoV-2 gains entry into host cells through the human angiotensin-converting enzyme 2 (ACE2) receptor, expressed in many tissues, including those in the cardiovascular system [6]. Upon binding to ACE2, the virus disrupts the receptor’s normal physiological roles, including its regulatory effects on the renin–angiotensin–aldosterone system (RAAS). This disruption can lead to increased angiotensin II levels, promoting vasoconstriction, inflammation and oxidative stress. Moreover, proinflammatory cytokines (such as IL-1β and type I and III interferons) released during severe COVID-19 can upregulate ACE2 expression, resulting in enhanced viral replication [7,8]. The resultant endothelial dysfunction and heightened systemic inflammation contribute to myocardial injury, manifesting as myocarditis or the exacerbation of pre-existing cardiovascular conditions. Notably, cardiac infection by SARS-CoV-2 has been frequently observed in autopsy cases [9]. Additionally, a meta-analysis by Yanbin Du et al. demonstrated that patients with hypertension had a 1.82-fold higher risk of developing critical COVID-19 and a 2.17-fold higher risk of COVID-19-related mortality [10]. These mechanisms collectively underscore the potential for SARS-CoV-2 to induce vascular complications, including thromboembolism and arrhythmias, further complicating disease prognosis.

### 1.2. SARS-CoV-2 Infection: Multifactorial Mechanisms Driving Arrhythmias

Numerous studies have established a correlation between SARS-CoV-2 infection and an increased incidence of arrhythmias across populations, including adults, children and adolescents [3,11,12,13]. These arrhythmias arise from multiple pathological mechanisms at the cellular, metabolic and pharmacological levels, encompassing myocardial hypoxia, hypoperfusion, the heightened secretion of proinflammatory cytokines and electrolyte imbalances. Contributing factors also include QT interval-prolonging medications and pre-existing conditions such as ischemic heart disease, severe heart failure, diabetes and obesity, as well as intensive care unit hospitalization [3,11].

Evidence indicates that arrhythmias occur more frequently in patients hospitalized with COVID-19 compared to those with pneumonia from other causes. Moreover, SARS-CoV-2-related arrhythmias have been linked to elevated mortality rates, with cardiac arrhythmias contributing to death in 20% of hospitalized patients [1,13]. The virus is also implicated in myocarditis, pericarditis and venous thromboembolism, underscoring its significant cardiovascular impact [11,14,15].

Supraventricular arrhythmias, commonly seen during SARS-CoV-2 infection, are more prevalent among individuals in their 5th and 6th decades of life with elevated inflammatory markers. These patients frequently experience hemodynamic instability, often necessitating intensive care [16,17].

### 1.3. COVID-19 Vaccination: Efficacy, Safety and Implications for Arrhythmias

Since the onset of the pandemic, extensive efforts have been made to develop effective antiviral therapies [18]. Although no definitive causal treatment exists, several vaccines have been developed, approved by the World Health Organization and widely implemented [11]. Vaccination has proven to significantly reduce infection rates and mortality among COVID-19 patients, with the benefits outweighing the potential side effects [11,19].

While some concerns have been raised regarding a potential association between COVID-19 vaccination and arrhythmias, large-scale studies have yet to confirm this link. The reported incidence remains rare, ranging from 1 to 76 cases per 10,000 vaccinated individuals [18].

### 1.4. Objective

Given the reported cardiovascular complications of SARS-CoV-2 infection and the ongoing debate regarding the arrhythmogenic effects of COVID-19 vaccination, this study aims to determine whether vaccination may serve as a protective factor against post-COVID-19 arrhythmias. We hypothesize that vaccinated individuals who have recovered from COVID-19 will exhibit a lower prevalence of arrhythmias compared to their unvaccinated counterparts, independent of other cardiovascular risk factors.

## 2. Material and Methods

The project examined patients hospitalized in the Department of Internal Medicine, Occupational Diseases, Hypertension and Clinical Oncology of the Wroclaw Medical University or consulted in the outpatient cardiology clinic to verify suspected cardiovascular diseases or, in the case of patients with diagnosed cardiovascular disease, to assess the effectiveness and possible optimization of its treatment and to assess its cardiovascular consequences. The criteria for qualifying patients for the study were as follows: age over 18 years, clinical indications for 24 h Holter monitoring and willingness to participate in the study. The criteria for exclusion from the study were as follows: previous cardiac surgery and vascular surgery; the presence of implantable devices (pacemakers/cardioverter-defibrillators); diagnosed neoplastic diseases and hematological, rheumatological and neurodegenerative diseases; ambiguous or incomplete data on history of SARS-CoV2 infection; and ambiguous or incomplete data on COVID-19 vaccination status. COVID-19 was confirmed by a PCR test. The study group of patients therefore included patients who had not been vaccinated against COVID-19 at all or patients who had completed the whole COVID-19 vaccination schedule.

Overall, the study group consisted of 237 patients (118 men and 119 women). The average age of the study group was 59.68 ± 11.30 years old. Most patients were diagnosed with arterial hypertension (*n* = 141, 59.5%). Furthermore, a considerable number of subjects were declared as habitual smokers (*n* = 41, 17.3%). We present the clinical characteristics of the entire study group in Table 1.

The study group consisted of patients who had previously had COVID-19, whereas the control group (C, *n* = 66) comprised patients with no history of COVID-19 infection. Additionally, the study group was subdivided into two groups: patients who were unvaccinated (A, *n* = 91) and vaccinated (B, *n* = 80) for COVID-19. According to the data shown in Table 2, there were no significant differences in terms of general characteristics either between groups A and B or between the study group (A, B) and the control group (C). In the case of COVID-19, patients not vaccinated against COVID-19 (group A) were hospitalized more often than patients vaccinated against COVID-19 (group B). However, the duration of hospitalization due to COVID-19 did not differ between these groups.

The participants filled out the informed consent form to participate in the study and were subsequently provided with a detailed description of the procedures. In the initial phase of the research, the participants were asked to fill out a medical questionnaire. Height and weight were measured and then body mass index (BMI) was calculated as weight in kilograms divided by the square of height in meters. Lipid metabolism parameters and fasting glucose levels as well as CRP were determined in blood. Twenty-four-hour Holter ECG analysis was analyzed by cardiologists skilled in Holter monitoring. The values of each parameter were presented as its mean value with standard deviation.

The statistical analysis was performed using the “STATISTICA 13” application (StatSoft, Krakow, Poland). Quantitative variables were presented as their arithmetic mean (X) and standard deviation (SD). The distribution of variables was verified with the W Shapiro–Wilk test. For quantitative variables with a normal distribution, one-way ANOVA was used in the comparative analysis. For such non-normally distributed variables, Kruskal–Wallis ANOVA was used. The Newman–Keuls test was used in the post hoc analysis. Qualitative variables were presented as percentages, and their comparative analysis was performed using the chi-square test. The relationship analysis consisted of regression analysis. Regression models were created using multivariate least squares analysis. Results at the *p* < 0.05 level were deemed statistically significant [20].

## 3. Results

Twenty-four-hour Holter monitoring was performed on the entire study group to register the following results, presented in Table 3. The mean heart rate was 73.14 ± 8.98, while the minimal heart rate equaled 51.17 ± 7.98 and the maximal heart rate equaled 115.55 ± 19.02. ECG monitoring recorded a notable number of ventricular (VE = 290.79 ± 92.21) and supraventricular (SVE = 372.32 ± 127.11) extrasystoles. In comparison, other arrhythmias appeared in lesser numbers, as is shown in Table 3.

According to the data in Table 4, there was a significant correlation between the occurrence of cardiac arrhythmias and COVID-19 vaccination history in patients with previous COVID-19 infection. Ventricular extrasystoles (VEs) were distinctively more common in patients, who had COVID-19 infection and were not vaccinated for COVID-19 (A) compared to the control group (C) (A = 617.48 ± 234.40 vs. C = 76.40 ± 18.12). Similarly, the research showed that supraventricular extrasystoles (SVEs) occurred remarkably more frequently in both unvaccinated (A) and vaccinated (B) patients after COVID-19 infection in relation to the control group (C) (A = 660.89 ± 324.36 vs. C = 138.96 ± 43.45; B = 236.61 ± 88.99 vs. C = 138.96 ± 43.45). In addition, the incidence of other arrhythmias varied among the groups; however, the differences were statistically irrelevant (*p* < 0.05).

Using backward stepwise multiple regression analysis, an attempt was made to determine independent risk factors for the occurrence of higher VE and SVE rates in 24 h Holter ECG monitoring. The potential risk factors were, each time, considered to be anthropometric parameters (age, sex, BMI), laboratory test results (total cholesterol, triglycerides, fasting glucose, CRP), smoking, CVD diseases (arterial hypertension, previous myocardial infarction, stroke, type 2 diabetes, venous thrombosis, atrial fibrillation), the use of antihypertensive drugs (diuretics, β-blockers, ACE inhibitors, sartans, Ca-blockers), hypoglycemic drugs (oral drugs, insulin), lipid-lowering drugs (statins, fibrates) and parameters characterizing COVID-19 (symptomatic COVID-19, lack of vaccination against COVID-19, hospitalization due to COVID-19 and the duration of this hospitalization). A regression analysis yielded the following models:

VE = 176.948 obesity + 201.347 arterial hypertension − 200.478 β-blockers + 187.158 myocardial infarction + 205.455 lack of vaccination against COVID-19.

SVE = 105.420 obesity + 312.048 diabetes type 2 + 125.148 lack of vaccination against COVID-19 − 151.274 β-blockers − 146.186 ACE inhibitors.

The obtained models demonstrate that, in the whole study group, obesity, arterial hypertension, previous myocardial infarction and lack of vaccination against COVID-19 are independent risk factors for higher VE rates. Obesity, diabetes type 2 and lack of vaccination against COVID-19 are independent risk factors for higher SVE rates. The use of β-blockers is an independent protective factor against higher VE and SVE rates, and the use of ACE inhibitors against higher SVE rates. The results of the regression analysis for the entire study group are shown in Table 5.

## 4. Discussion

Our study revealed significant findings concerning the relationship between COVID-19 infection, vaccination status and cardiac arrhythmias. Specifically, we observed that ventricular extrasystoles (VEs) and supraventricular extrasystoles (SVEs) occurred more frequently in unvaccinated patients with a history of COVID-19 infection compared to vaccinated individuals and controls. Regression analysis identified lack of vaccination, obesity and comorbid conditions such as hypertension and diabetes as independent predictors of increased arrhythmias. These results highlight the protective role of COVID-19 vaccination in mitigating severe cardiac manifestations and provide valuable insights into risk stratification for post-COVID-19 cardiac care. Additionally, the identified predictors align with the broader understanding of arrhythmogenic risk factors, emphasizing the need for targeted preventive measures in high-risk populations. These findings align with the growing body of evidence demonstrating the cardiovascular impact of SARS-CoV-2 infection, as detailed in previous studies [21,22].

Numerous advanced research studies on patients with COVID-19 have been conducted since 2020 in order to gather data on management, possible complications and treatment [23]. While our understanding of SARS-CoV-2 has improved, revealing its wide-ranging impact at both the cellular and pathophysiological levels, new discoveries continue to unveil previously unknown complications. Initially, researchers focused primarily on pulmonary complications, such as acute respiratory failure [2]. Subsequent studies, however, demonstrated that dysfunction in the cardiovascular system is a significant component of the natural history of COVID-19 [6,12,24]. The cardiological complications of COVID-19 can be categorized into five groups, including (a) myocardial injury (mainly due to ischemia or myocarditis); (b) arrhythmias; (c) the development or exacerbation of heart failure; (d) venous thromboembolism; and (e) cardiac treatment complications [15,25]. Beyond these well-established cardiovascular manifestations, recent reports have highlighted the occurrence of COVID-19-related multisystem thrombosis and intracardiac thrombosis, further underscoring the hypercoagulable state induced by the virus. Garg et al. [26] documented cases of left ventricular mural thrombi in patients with myocardial injury during COVID-19, whereas Sonaglioni et al. [27] described an unusual case of biventricular thrombosis in a patient with ischemic dilated cardiomyopathy, emphasizing the embolic risks associated with these thrombotic events.

The occurrence of diverse supraventricular and ventricular arrhythmias has been unequivocally proven by multiple studies [2,11,28,29]. Liao et al. demonstrated arrhythmias in 16% of patients with COVID-19, including 12% non-classified arrhythmias, 8.2% atrial fibrillation, 10.8% conduction disorders, 8.6% premature systoles and 3.3% ventricular fibrillation or ventricular tachycardia [13]. Similarly, another study confirmed arrhythmias in patients with COVID-19. Among 683 respondents, 21% were diagnosed with atrial fibrillation, 5.4% with atrial flutter, 3.5% with sustained atrial tachycardia and 5.7% with paroxysmal supraventricular tachycardia [30]. Additionally, Pranata et al. analyzed four studies featuring 784 patients and concluded that the prevalence of arrhythmia in patients with COVID-19 was 19%. According to Zhan et al., the prevalence of arrhythmia in patients with COVID-19 oscillates between 10% and 20% [2]. Our observation of increased VE and SVE rates in unvaccinated patients mirrors the trends reported by Patone et al. [31], emphasizing the arrhythmogenic risks in this subgroup. On the other hand, according to Manolis et al., the probability of arrhythmia, including life-threatening ventricular arrhythmias, depends to a considerable degree on the patient’s general condition, their medical history, comorbid conditions and already applied chronic treatment [28]. In patients with an elevated troponin T level (cTnT), ventricular arrhythmias were more likely to develop (17.3%), including ventricular fibrillation in 5.9% of patients.

Emerging evidence highlights the interplay between myocardial injury and cardiovascular outcomes in COVID-19. SARS-CoV-2 infection can precipitate myocardial damage via multiple mechanisms, including direct viral invasion, systemic inflammation and pre-existing cardiovascular conditions. According to Patone et al. [31], both SARS-CoV-2 infection and COVID-19 vaccination are associated with risks of myocarditis and arrhythmias, but the severity of cardiac injury is notably higher in SARS-CoV-2-infected individuals. Elevated biomarkers such as troponin and NT-pro-BNP are closely correlated with myocardial injury severity and mortality in these patients [31].

Manolis et al. [28] further elucidated the arrhythmogenic potential of COVID-19, demonstrating that inflammatory cytokines and hypoxia contribute to myocardial excitability. These changes heighten the risk of life-threatening arrhythmias in critically ill patients [28]. This is supported by evidence from Cersosimo et al. [32] and Kapusta et al. [33], who identified elevated cardiac biomarkers as independent predictors of mortality. Their findings underscore the prognostic importance of these markers in stratifying high-risk patients and guiding therapeutic interventions [32].

Abdel Moneim et al. [34] reviewed the multifactorial mechanisms underlying cardiovascular manifestations in COVID-19. They highlighted that systemic inflammation, coagulopathy and electrolyte imbalances exacerbate myocardial damage. This aligns with our findings of increased arrhythmia prevalence in patients with elevated troponin levels, reinforcing the link between systemic inflammation and cardiac outcomes. Moreover, comorbid conditions like hypertension amplify susceptibility to myocardial injury, as reported in multiple studies [15,34].

Cersosimo et al. [32] also emphasized that monitoring NT-pro-BNP levels in hospitalized patients provides critical insights into disease progression and prognosis. Patients with persistently high biomarker levels often exhibit worse outcomes, reinforcing the importance of early cardiovascular evaluation in COVID-19 management [32].

Knowledge of these facts is crucial because cardiovascular diseases correlate with a poorer prognosis [35]. The major group of patients with cardiological complications is likely to require hospitalization in the intensive care unit, the implementation of catecholamine vasopressors and electrical cardioversion [36]. In some patients, SARS-CoV-2 infection might cause severe ventricular arrhythmias, ultimately leading to the patient’s death [16].

Our findings highlight the need for continued cardiovascular monitoring in individuals recovering from COVID-19, particularly those with pre-existing cardiovascular risk factors or a history of severe infection. Emerging evidence suggests that SARS-CoV-2 infection may lead to persistent vascular dysfunction, endothelial damage and an increased risk of long-term cardiovascular complications, including arrhythmias [37]. These alterations, which may contribute to prolonged arrhythmogenic risk, myocardial fibrosis and autonomic dysregulation, underscore the potential benefit of regular long-term ECG monitoring in post-COVID-19 patients. Future studies should focus on longitudinal follow-up to better characterize these long-term effects and establish evidence-based recommendations for post-COVID-19 cardiac care.

It is important to acknowledge that the observed protective effect of COVID-19 vaccination against arrhythmias may, at least in part, be influenced by selection bias. Vaccinated individuals may have better access to healthcare, higher health awareness and healthier lifestyle behaviors compared to those who remained unvaccinated [38]. These factors could contribute to lower cardiovascular risk independent of the direct effects of vaccination. Future studies should consider adjusting for these potential confounders to ensure a more precise assessment of the vaccine’s role in mitigating post-COVID-19 arrhythmic events.

## 5. Conclusions

Our current understanding of the antiarrhythmic advantages of COVID-19 vaccination is still limited and requires further exploration. Since the beginning of the pandemic, researchers have been intensively analyzing the risk of arrhythmic incidents during and after SARS-CoV-2 infection. However, only a few studies have been conducted to prove the correlation between COVID-19, vaccination and arrhythmia occurrence in patients. In this study, the authors obtained promising results for the future, facilitating further discussions and research on the topic of the antiarrhythmic advantages of COVID-19 vaccination. Moreover, the knowledge acquired in this study serves as a valuable tool for effectively promoting COVID-19 vaccination among patients. The proven advantages, including its preventive influence on heart function in the context of arrhythmia prevalence during and after infection, significantly outweigh the possible adverse effects.

## Figures and Tables

**Table 1:** Clinical characteristics of entire study group (*n* = 237).

	Study Group (*n* = 237)
age (years)	59.68 ± 11.30
sex (%/n)	
men	49.8/118
female	50.2/119
height (cm)	171.14 ± 12.38
body weight (kg)	76.82 ± 9.27
BMI (kg/m^2^)	26.14 ± 6.31
arterial hypertension (%/n)	59.5/141
systolic blood pressure (mmHg)	138.10 ± 23.67
diastolic blood pressure (mmHg)	83.78 ± 12.85
diuretics (%/n)	27.8/66
β-blockers (%/n)	33.3/79
ACE inhibitors (%/n)	35.0/83
sartans (%/n)	10.5/25
Ca-blockers (%/n)	27.0/64
total cholesterol (mg/dL)	165.31 ± 39.10
triglicerides (mg/dL)	124.29 ± 68.07
statins (%/n)	59.1/140
fibrates (%/n)	19.4/46
miocardial infarction (%/n)	8.4/20
stroke (%/n)	2.9/7
atrial fibrillation (%/n)	5.1/12
deep vein thrombosis (%/n)	3.3/8
diabetes type 2 (%/n)	11.8/28
fasting glucose (mg/dL)	103.31 ± 15.44
oral hypoglycemic drugs (%/n)	11.8/28
insulin (%/n)	4.6/11
thyroid diseases (%/n)	8.4/20
smoking (%/n)	17.3/41
cigarette years	274.23 ± 148.52
CRP (mg/L)	2.20 ± 4.97

BMI—body mass index; CRP—C-reactive protein.

**Table 2:** Clinical characteristics in studied subgroups.

	Group A (*n* = 91)	Group B (*n* = 80)	Group C (*n* = 66)	*p* < 0.05
age (years)	59.75 ± 14.82	62.11 ± 8.95	60.53 ± 10.28	ns
sex (%/n)				
men	51.6/47	46.2/37	51.5/34	ns
female	48.4/44	53.7/43	48.5/32	ns
height (cm)	172.52 ± 11.18	170.52 ± 8.83	168.14 ± 9.27	ns
body weight (kg)	77.63 ± 11.48	75.28 ± 8.26	75.59 ± 12.15	ns
BMI (kg/m^2^)	27.83 ± 2.92	26.17 ± 2.27	25.26 ± 3.59	ns
arterial hypertension (%/n)	49.4/45	70.0/56	60.6/40	ns
systolic blood pressure (mmHg)	137.58 ± 25.79	140.31 ± 20.83	136.14 ± 23.98	ns
diastolic blood pressure (mmHg)	82.75 ± 12.96	85.44 ± 12.81	83.18 ± 12.76	ns
diuretics (%/n)	23.1/21	35.0/28	25.8/17	ns
β-blockers (%/n)	26.4/24	41.2/33	33.3/22	ns
ACE inhibitors (%/n)	27.5/25	42.5/34	36.4/24	ns
sartans (%/n)	9.9/9	11.2/9	10.6/7	ns
Ca-blockers (%/n)	24.2/22	26.2/21	31.8/21	ns
total cholesterol (mg/dL)	163.34 ± 39.44	163.59 ± 40.14	170.10 ± 37.48	ns
triglicerides (mg/dL)	128.30 ± 64.97	116.24 ± 68.20	128.52 ± 72.16	ns
statins (%/n)	59.3/54	57.5/46	60.6/40	ns
fibrates (%/n)	20.88/19	18.75/15	18.2/12	ns
myocardial infarction (%/n)	8.8/8	8.7/7	7.6/5	ns
stroke (%/n)	3.3/3	2.5/2	3.0/2	ns
atrial fibrillation (%/n)	5.5/5	5.0/4	4.5/3	ns
deep vein thrombosis (%/n)	4.4/4	3.7/3	1.5/1	ns
diabetes type 2 (%/n)	9.9/9	15.0/12	10.6/7	ns
oral hypoglycemic drugs (%/n)	9.9/9	15.0/12	10.6/7	ns
insulin (%/n)	5.5/5	5.0/4	3.0/2	ns
fasting glucose (mg/dL)	103.81 ± 16.49	105.42 ± 16.88	100.05 ± 11.22	ns
thyroid diseases (%/n)	8.8/8	8.7/7	7.6/5	ns
smoking (%/n)	16.5/15	17.5/14	18.2/12	ns
cigarette years	272.19 ± 152.86	272.00 ± 146.85	284.64 ± 153.38	ns
CRP (mg/L)	2.09 ± 4.70	2.21 ± 4.96	2.35 ± 5.40	ns
hospitalization due to COVID-19 (%/n)	56.0/51	20.0/16.0	-	A vs. B
duration of hospitalization due to COVID-19 (days)	21.63 ± 15.64	22.69 ± 17.98	-	ns
vaccine against COVID-19 (%/n)				
Comirnaty (Pfizer-BioNTech)	0.0/0	80.0/64	78.8/52	A vs. B; A vs. C
Spikevax (Moderna, NIAID)	0.0/0	13.7/11	9.1/6	ns
Vaxzevria (AstraZeneca)	0.0/0	9.1/6	4.5/3	ns
lack of vaccination	100.0/91	0.0/0	7.6/5	A vs. B; A vs. C

BMI—body mass index; CRP—C-reactive protein; ns—not statistically significant; A, B, C are the names of the groups.

**Table 3:** Parameters of 24 h Holter ECG monitoring for entire study group (*n* = 237).

	Study Group (*n* = 237)
HR Min (bpm)	51.17 ± 7.98
HR max (bpm)	115.55 ± 19.02
HR mean (bpm)	73.14 ± 8.98
VEs	290.79 ± 92.21
SVEs	372.32 ± 127.11
bradycardia (number)	28.87 ± 10.35
minimum bradycardia	40.51 ± 6.73
tachycardia (number)	27.46 ± 18.79
maximum tachycardia	151.67 ± 18.79
VT	0.13 ± 0.07
SVT	2.58 ± 1.47
AF	0.18 ± 0.35
ventricular rhythm	0.08 ± 0.05

HR max—maximum heart rate; HR mean—average heart rate; HR min—minimum heart rate; VEs—ventricular extra beats; SVEs—supraventricular beats; VT—ventricular tachycardia; SVT—supraventricular tachycardia; AF—atrial fibrillation.

**Table 4:** Parameters of 24 h Holter ECG monitoring in studied subgroups.

	Group A (*n* = 91)	Group B (*n* = 80)	Group C (*n* = 66)	*p* < 0.05
HR min (bpm)	52.16 ± 7.20	51.68 ± 5.62	50.73 ± 9.43	ns
HR max (bpm)	122.66 ± 18.92	115.27 ± 20.27	113.20 ± 17.06	ns
HR mean (bpm)	74.21 ± 9.07	73.84 ± 10.19	72.11 ± 8.46	ns
VEs	617.48 ± 234.40	96.05 ± 36.57	76.40 ± 18.12	A vs. C
SVEs	660.89 ± 324.36	236.61 ± 88.99	138.96 ± 43.45	A vs. C; B vs. C
bradycardia (number)	32.34 ± 15.58	35.13 ± 11.38	22.63 ± 10.46	ns
minimum bradycardia	40.57 ± 6.75	41.38 ± 7.52	40.68 ± 6.87	ns
tachycardia (number)	35.52 ± 24.79	25.24 ± 10.18	14.52 ± 5.2	ns
maximum tachycardia	149.22 ± 17.02	153.57 ± 20.12	147.98 ± 20.11	ns
VT	0.16 ± 0.07	0.14 ± 0.09	0.02 ± 0.01	ns
SVT	3.86 ± 1.87	1.11 ± 0.45	0.47 ± 0.98	ns
AF	0.45 ± 0.41	0.05 ± 0.15	0.00 ± 0.00	ns
ventricular rhythm	0.11 ± 0.10	0.04 ± 0.02	0.00 ± 0.00	ns

HR max—maximum heart rate; HR mean—average heart rate; HR min—minimum heart rate; VEs—ventricular extra beats; SVEs—supraventricular beats; VT—ventricular tachycardia; SVT—supraventricular tachycardia; AF—atrial fibrillation; ns—not statistically significant; A, B, C are the names of the groups.

**Table 5:** Results of regression analysis for entire study group.

	Model for VEs			Model for SVEs		
	Regression Coefficient	SEM of Regression Coefficient	*p*	Regression Coefficient	SEM of Regression Coefficient	*p*
obesity	176.948	72.635	0.035	105.420	46.678	0.041
arterial hypertension	201.347	102.098	0.037	-	-	-
β-blockers	−200.478	85.852	0.028	−151.274	60.534	0.018
ACE inhibitors	-	-	-	−146.186	42.425	0.026
myocardial infarction	187.158	71.255	0.035	-	-	-
diabetes type 2	-	-	-	312.048	199.274	0.043
lack of vaccination against COVID-19	205.455	94.356	0.038	125.148	40.126	0.033

VEs—ventricular extra beats; SEM—standard error of mean; SVEs—supraventricular beats.

## Data Availability

The data presented in this study are available on request from the corresponding author.
